# SARAF and EFHB Modulate Store-Operated Ca^2+^ Entry and Are Required for Cell Proliferation, Migration and Viability in Breast Cancer Cells

**DOI:** 10.3390/cancers13164160

**Published:** 2021-08-19

**Authors:** Isaac Jardin, Joel Nieto-Felipe, Sandra Alvarado, Raquel Diez-Bello, Jose J. Lopez, Ginés M. Salido, Tarik Smani, Juan A. Rosado

**Affiliations:** 1 Department of Physiology, Institute of Molecular Pathology Biomarkers, University of Extremadura, 10003 Caceres, Spain; jonietof@alumnos.unex.es (J.N.-F.); sandraam@unex.es (S.A.); raqueldiez@unex.es (R.D.-B.); jjlopez@unex.es (J.J.L.); gsalido@unex.es (G.M.S.); 2 Department of Medical Physiology and Biophysic, Institute of Biomedicine of Sevilla, 41013 Sevilla, Spain; tasmani@us.es

**Keywords:** EFHB, SARAF, breast cancer cells, viability, migration, calcium entry

## Abstract

**Simple Summary:**

Breast cancer accounts as the most extended disease among women worldwide. Store-operated calcium entry (SOCE), a major mechanism that allows calcium entry from the extracellular region through the plasma membrane, is required for several physiological processes. In recent years, it has been revealed that several breast cancer types present dysregulated calcium homeostasis, which contribute to their malignancy. Here we show the role of two important regulators of SOCE, SARAF and EFHB, which are necessary for cell viability, proliferation, and migration in breast cancer and pre-neoplastic cells, respectively, thus suggesting that these regulators play a key function in breast cancer development and progression.

**Abstract:**

Breast cancer is among the most common malignancies in women. From the molecular point of view, breast cancer can be grouped into different categories, including the luminal (estrogen receptor positive (ER+)) and triple negative subtypes, which show distinctive features and, thus, are sensitive to different therapies. Breast cancer cells are strongly dependent on Ca^2+^ influx. Store-operated Ca^2+^ entry (SOCE) has been found to support a variety of cancer hallmarks including cell viability, proliferation, migration, and metastasis. The Ca^2+^ channels of the Orai family and the endoplasmic reticulum Ca^2+^ sensor STIM1 are the essential components of SOCE, but the extent of Ca^2+^ influx is fine-tuned by several regulatory proteins, such as the STIM1 modulators SARAF and EFHB. Here, we show that the expression and/or function of SARAF and EFHB is altered in breast cancer cells and both proteins are required for cell proliferation, migration, and viability. EFHB expression is upregulated in luminal and triple negative breast cancer (TNBC) cells and is essential for full SOCE in these cells. SARAF expression was found to be similar in breast cancer and pre-neoplastic breast epithelial cells, and SARAF knockdown was found to result in enhanced SOCE in pre-neoplastic and TNBC cells. Interestingly, silencing SARAF expression in ER+ MCF7 cells led to attenuation of SOCE, thus suggesting a distinctive role for SARAF in this cell type. Finally, we used a combination of approaches to show that molecular knockdown of SARAF and EFHB significantly attenuates the ability of breast cancer cells to proliferate and migrate, as well as cell viability. In aggregate, SARAF and EFHB are required for the fine modulation of SOCE in breast cancer cells and play an important role in the maintenance of proliferation, migration, and viability in these cells.

## 1. Introduction

Store-operated Ca^2+^ entry (SOCE), a mechanism for Ca^2+^ influx regulated by the filling state of the intracellular Ca^2+^ stores, is involved in the activation of cellular functions ranging from secretion to gene transcription, and dysregulation of this pathway has been associated to different disorders, including cancer [1]. Cancer cells are characterized by shifting the cell cycle control towards enhanced proliferation while suppressing cell death [2]. The mechanisms underlying these changes involve up- or downregulation of a variety of Ca^2+^ channels and regulatory proteins, including those involved in SOCE. The essential elements of SOCE are the Ca^2+^ release-activated Ca^2+^ (CRAC) channels, consisting of homo- or heterohexamers of Orai proteins [3,4], and the endoplasmic reticulum Ca^2+^ sensors, STIM1 and STIM2 [5,6,7,8]. Breast cancer is among the most common cancer types in women. In breast cancer cells, Orai1 and TRPC6 are upregulated in the estrogen receptor positive (ER+) and triple negative subtypes, and Orai3 is overexpressed exclusively in the ER+ breast cancer subtype [9,10]. SOCE through (or facilitated by) these channels has been reported to be critical for the development of a variety of breast cancer hallmarks, including cell proliferation, migration, and metastasis [9,10,11,12].

In addition to the key molecular players, a variety of regulatory proteins contribute to fine tune SOCE to match the Ca^2+^ responses to the strength of agonist stimulation. Among the SOCE regulatory proteins, SARAF has been identified as a negative STIM1 modulator that protects cells from Ca^2+^ overload [13]. SARAF is a 339-amino acid transmembrane protein that interacts with STIM1 at rest to prevent spontaneous activation of SOCE, but has also been found to be responsible for slow Ca^2+^-dependent inactivation (SCDI) of CRAC channels [13,14]. SARAF is predominantly expressed in the endoplasmic reticulum (ER) [13] but a pool is constitutively expressed in the plasma membrane where SARAF negatively modulates a Ca^2+^ influx evoked by arachidonate [15]. SARAF has also been found to modulate the activity of Orai1 and TRPC1 in a STIM1-independent manner, potentiating Ca^2+^ influx through the former [16] and playing a negative regulatory role in TRPC1-mediated Ca^2+^ entry [17]. In neuroblastoma cells, SARAF has been reported to modulate the ability of arachidonate to promote cell survival [15]. Furthermore, SARAF interacts with Orai1 in endothelial cells and contributes to the activation of angiogenesis [18]. SARAF interacts with the STIM1 SOAR region by a mechanism modulated by the SOAR C-terminal inhibitory domain (CTID). CTID is located downstream the SOAR region and consists of two lobes: the N-terminal CTID lobe (aa 448–490), which restricts the STIM1-SARAF interaction, and the C-terminal lobe (aa 490–530), which facilitates the interaction [14].

CRAC channel inactivation by SARAF is impaired by cell loading with the fast intracellular Ca^2+^ chelator BAPTA, thus indicating that the role of SARAF depends on the presence of free cytosolic Ca^2+^ [13]; however, SARAF does not contain Ca^2+^-binding domains. We recently identified an EF-hand containing protein, an EF-hand domain family member B (EFHB), which might act as a Ca^2+^ sensor regulating the interaction between STIM1 and SARAF. EFHB is an 833-amino acid cytosolic protein that exhibits two EF-hand domains and is essential for the STIM1–Orai1 interaction and full SOCE activation. Upon Ca^2+^ store depletion, EFHB interacts with STIM1 and displaces SARAF, thus allowing STIM1 to interact with and activate CRAC channels in the plasma membrane. Then, a Ca^2+^ influx induces the dissociation of STIM1 from EFHB and its re-association with SARAF to mediate SCDI [19]. In the interactive database (www.proteinatlas.org, accessed on 18 June 2021), both EFHB and SARAF proteins, but mostly the latter, are detected at the RNA level in breast tissue and breast cancer samples, but protein expression has not been analyzed.

In the present study, we have investigated the role of SARAF and EFHB in SOCE in ER+ and triple negative breast cancer (TNBC) cells. Here, we show that SARAF and EFHB are expressed in neoplastic and pre-neoplastic breast cells, with breast cancer cells showing upregulation of EFHB expression. Both proteins play an important role in SOCE in neoplastic and pre-neoplastic cells and are essential for breast cancer cell survival, proliferation, and migration. These findings provide new insights into the mechanism regulating Ca^2+^ influx in breast cancer cells and its functional role.

## 2. Results

### 2.1. Expression of EFHB and SARAF, and Functional Role in SOCE, in Breast Cancer and Pre-Neoplastic Epithelial Cells

**Figure 1:**
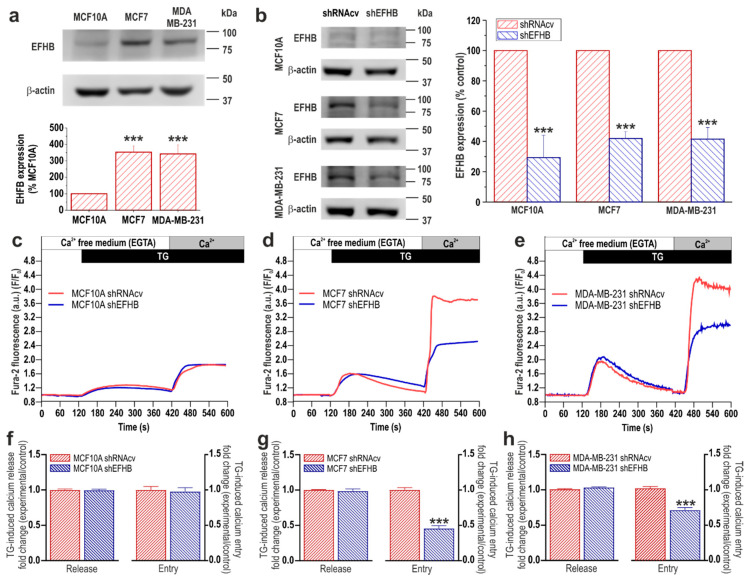
EFHB is expressed in breast cancer and pre-neoplastic epithelial cells and plays a relevant role in SOCE. (**a**) MCF10A, MCF7, and MDA-MB-231 cells were lysed and analyzed using Western blotting with a specific anti-EFHB antibody. The membrane was reprobed with an anti-β-actin antibody for protein loading control. Bar graphs represent EFHB expression normalized to the β-actin content and expressed as percentage of the expression in MCF10A cells. Molecular masses indicated on the right were determined using molecular-mass markers run in the same gel. *** *p* < 0.001 compared to EFHB expression in MCF10A cells. (**b**) MCF10A, MCF7, and MDA-MB-231 cells were transfected with shEFHB or scramble plasmids (shRNAcv), as indicated. After 48 h cells were lysed and analyzed using Western blotting with an anti-EFHB antibody, followed by reprobing with an anti-β-actin antibody for protein loading control. Bar graphs represent EFHB expression normalized to the β-actin content at the different experimental conditions and expressed as percentage of the expression in shRNAcv-treated cells (control). *** *p* < 0.001 compared to EFHB expression in shRNAcv-treated cells. Uncropped Western blot figures in Figure S2. (**c**–**h**) MCF10A, MCF7, and MDA-MB-231 cells were transfected with shEFHB or scramble plasmids (shRNAcv), as indicated. Forty-eight hours after transfection, cells were loaded with fura-2 and perfused with a Ca^2+^-free medium (100 µM of EGTA was added). Cells were then stimulated with TG (2 µM) followed by the reintroduction of external Ca^2+^ (final concentration of 1 mM) to initiate Ca^2+^ entry. Bar graphs represent TG-induced Ca^2+^ release and entry in MCF10A (**f**), MCF7 (**g**) and MDA-MB-231 (**h**), expressed as fold change experimental over control (shRNAcv-treated cells). Data are mean ± SEM of 40 cells/day/3–5 days. *** *p* < 0.001 compared to Ca^2+^ entry in control cells.

**Figure 2:**
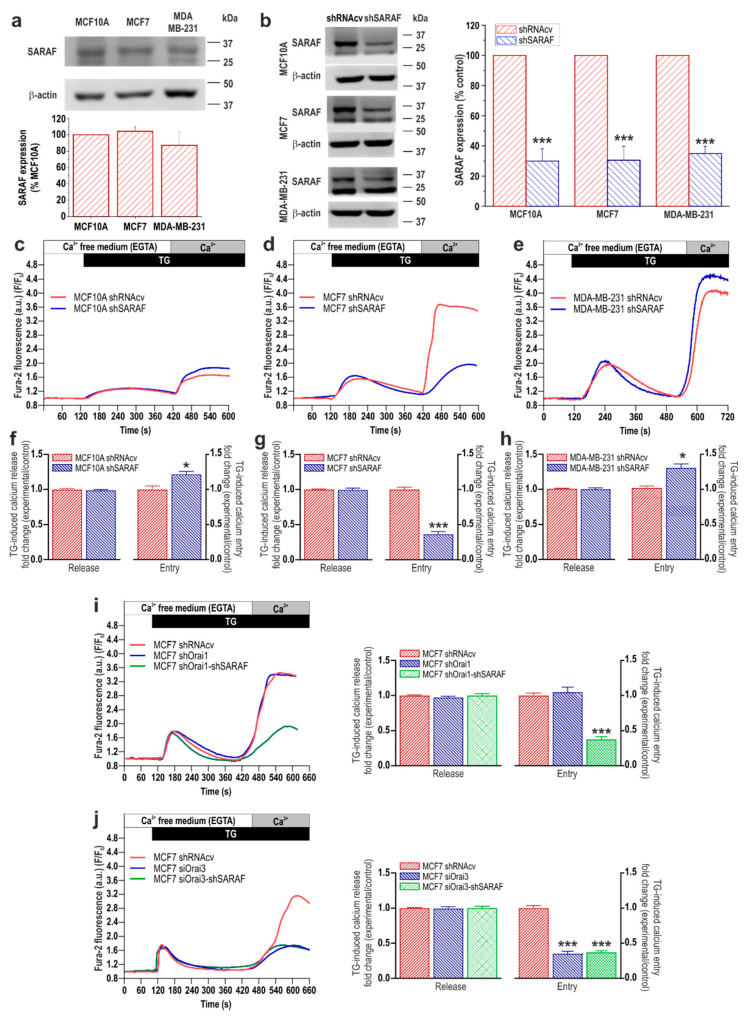
SARAF expression and functional role in SOCE in breast cancer and pre-neoplastic cells. (**a**) MCF10A, MCF7, and MDA-MB-231 cells were lysed and analyzed using Western blotting with a specific anti-SARAF antibody. The membrane was reprobed with an anti-β-actin antibody for protein loading control. Bar graphs represent SARAF expression normalized to the β-actin content and expressed as percentage of the expression in MCF10A cells. Molecular masses indicated on the right were determined using molecular-mass markers run in the same gel. (**b**) MCF10A, MCF7, and MDA-MB-231 cells were transfected with shSARAF or scramble plasmids (shRNAcv), as indicated. After 48 h cells were lysed and analyzed using Western blotting with an anti-SARAF antibody, followed by reprobing with an anti-β-actin antibody for protein loading control. Bar graphs represent SARAF expression normalized to the β-actin content at the different experimental conditions and expressed as percentage of the expression in shRNAcv-treated cells (control). *** *p* < 0.001 compared to SARAF expression in shRNAcv-treated cells. Uncropped Western blot figures in Figure S2. (**c**–**h**) MCF10A, MCF7. and MDA-MB-231 cells were transfected with shSARAF or scramble plasmids (shRNAcv), as indicated. Forty-eight hours after transfection, cells were loaded with fura-2 and perfused with a Ca^2+^-free medium (100 µM of EGTA was added). Cells were then stimulated with TG (2 µM) followed by reintroduction of external Ca^2+^ (final concentration 1 mM) to initiate Ca^2+^ entry. Bar graphs represent TG-induced Ca^2+^ release and entry in MCF10A (**f**), MCF7 (**g**) and MDA-MB-231 (**h**), expressed as fold change experimental over control (shRNAcv-treated cells). Data are mean ± SEM of 40 cells/day/3–5 days. * *p* < 0.05 and *** *p* < 0.001 compared to Ca^2+^ entry in control cells. (**i**,**j**) MCF7 cells were transfected with shOrai1 alone or in combination with shSARAF (**i**) or esiOrai3 alone or in combination with shSARAF (**j**) or scramble plasmids (shRNAcv), as indicated. Forty-eight hours after transfection, cells were loaded with fura-2 and perfused with a Ca^2+^-free medium (100 µM EGTA added). Cells were then stimulated with TG (2 µM) followed by reintroduction of external Ca^2+^ (final concentration 1 mM) to initiate Ca^2+^ entry. Bar graphs represent TG-induced Ca^2+^ release and entry in MCF7 cells, expressed as fold change experimental over control (shRNAcv-treated cells). Data are mean ± SEM of 40 cells/day/3–5 days. *** *p* < 0.001 compared to Ca^2+^ entry in control cells.

To investigate the possible functional role of EFHB and SARAF in breast neoplastic and pre-neoplastic cells, we first analyze the expression of these proteins in the pre-neoplastic breast epithelial MCF10A cell line as well as in the estrogen receptor positive (ER+) breast cancer MCF7 cell line and the triple negative breast cancer (TNBC) cell line MDA-MB-231. As shown in Figure 1a and Figure 2a, Western blot analysis of whole-cell lysates from the MCF10A, MCF7, and MDA-MB-231 cell lines with specific anti-EFHB (Figure 1) or anti-SARAF (Figure 2) antibodies revealed the expression of EFHB and SARAF in the cell lines investigated. Our results indicate that MCF10A expresses a low but detectable amount of EFHB but, interestingly, MCF7 and MDA-MB-231 cells exhibit a significantly greater expression (Figure 1a; *p* < 0.001 Kruskal–Wallis test combined with Dunn’s post hoc test; *n* = 6). By contrast, the expression of SARAF was found to be similar in neoplastic and pre-neoplastic cells (Figure 2a; *n* = 6).

SOCE in MCF7 and MDA-MB-231 cells has been reported to be strongly dependent on STIM1 [9]; therefore, we have further explored the functional role of the STIM1 modulators, EFHB and SARAF, in SOCE in these cell types, as compared to MCF10A breast epithelial cells. To assess the role of EFHB in SOCE, MCF10A, MCF7, and MDA-MB-231 cells were transfected with shRNA for EFHB [19] or scramble plasmids to analyze the effect of EFHB knockdown on thapsigargin (TG)-evoked Ca^2+^ entry. As shown in Figure 1b, transfection with shEFHB significantly attenuated the expression of the target protein by about 60–70% in 48 h (*p* < 0.001; Mann–Whitney U test). As depicted in Figure 1c, in cells transfected with scramble plasmids and suspended in a Ca^2+^-free medium, treatment with the SERCA inhibitor TG (2 µM) resulted in a transient increase in the fura-2 fluorescence ratio due to Ca^2+^ release from the intracellular Ca^2+^ stores. Subsequent addition of Ca^2+^ to the extracellular medium resulted in a rise in the fura-2 fluorescence ratio indicative of a Ca^2+^ influx. Cell transfection with the shEFHB plasmid did not induce any significant effect on the resting fura-2 fluorescence ratio (in ratio a.u.: MCF10A shRNAcv: 0.47 ± 0.08 vs. shEFHB: 0.48 ± 0.09; MCF7 shRNAcv: 0.33 ± 0.06 vs. shEFHB: 0.36 ± 0.07; MDA-MB-231 shRNAcv: 0.33 ± 0.06 vs. shEFHB: 0.35 ± 0.06) or the rise in the ratio induced by TG (Figure 1f–h), thus suggesting that EFHB knockdown does not alter the ability of the cells to accumulate Ca^2+^ into the ER. In addition, these findings suggest that transfection of the plasmid does not alter the Ca^2+^ leakage rate from the ER. EFHB knockdown does not significantly modify SOCE in MCF10A, which is consistent with the low expression of the protein in this cell type and indicates that EFHB does not play a relevant role in the activation of SOCE in MCF10A cells (Figure 1c,f; *n* = 40 cells/day/3–5 days). By contrast, attenuation of EFHB expression significantly reduced SOCE in breast cancer MCF7 and MDA-MB-231 cells (Figure 1d,e,g,h; *p* < 0.001 Student’s *t*-test; *n* = 40 cells/day/3–5 days). These findings indicate that EFHB plays a relevant role in the activation of SOCE in breast cancer cells.

Furthermore, we have evaluated the role of SARAF in SOCE in MCF10A cells and the cancer cell lines MCF7 and MDA-MB-231. To investigate this phenomenon, cells were transfected with shSARAF or scramble plasmids, and the effect of SARAF knockdown in SOCE was assessed by fluorescence microscopy. As shown in Figure 2b, transfection with shSARAF significantly reduced the expression of SARAF by about 60–70% in 48 h (*p* < 0.001; Mann–Whitney U test), depending on the cell type evaluated. Our results indicate that SARAF knockdown did not significantly modify either the resting fura-2 fluorescence ratio (in ratio a.u.: MCF10A shRNAcv: 0.47 ± 0.08 vs. shSARAF: 0.45 ± 0.06; MCF7 shRNAcv: 0.33 ± 0.06 vs. shSARAF: 0.35 ± 0.06; MDA-MB-231 shRNAcv: 0.33 ± 0.06 vs. shSARAF: 0.35 ± 0.07) or the rise in the Ca^2+^ signal induced by TG (Figure 2f–h). The latter suggests that SARAF knockdown does not modify the ability of these cells to accumulate Ca^2+^ into the ER, as previously described in SH-SY5Y [15] and MEG01 cells [16]; however, in HeLa cells, SARAF knockdown significantly enhanced the ER Ca^2+^ level [13]. SARAF knockdown significantly enhances SOCE in MCF10A and MDA-MB-231 cells (Figure 2c,e,f,h; *n* = 40 cells/day/3–5 days), which is compatible with a role of SARAF in cells where SOCE is predominantly mediated by STIM1 and Orai1 [13,16]. By contrast, attenuation of SARAF expression significantly reduced SOCE in breast cancer MCF7 cells, where SOCE is strongly dependent on STIM1, STIM2, and Orai3 [9] (Figure 2d,e,g,h; *p* < 0.05–0.001 Student’s *t* test; *n* = 40 cells/day/3–5 days).

As SARAF is a well-known inhibitor of STIM1 and STIM2 [13], we have further tested whether the effect of SARAF in SOCE and MCF7 cells is mediated by Orai1 or Orai3. To address this phenomenon, we transfected cells with shOrai1, and shOrai1, in combination with shSARAF or scramble plasmids and SOCE, was determined as described above. Our results indicate that Orai1 knockdown, alone or in combination with SARAF knockdown, did not significantly modify the resting fura-2 fluorescence ratio (MCF7 shRNAcv: 0.35 ± 0.05 vs. shOrai1: 0.34 ± 0.06 or shOrai1+shSARAF: 0.36 ± 0.06) or Ca^2+^ release from the intracellular stores (Figure 2i). As shown in Figure 2i, transfection of MCF7 cells with shOrai1 did not significantly modify SOCE but did so in combination with shSARAF-attenuated SOCE, to a similar extent whereby SARAF knockdown was carried out alone (Figure 2d,g; *p* < 0.001; *n* = 40 cells/day/3–5 days), which indicates that Orai1 is not involved in SOCE in MCF7 cells and that the role of SARAF in MCF7 cells is independent on Orai1. We have further evaluated the role of Orai3 on the SARAF-mediated response. Cells were transfected with esiOrai3 (a pool of siRNA that target the Orai3 mRNA), alone or in combination with shSARAF, or scramble plasmids and SOCE were determined as described above. Our results indicate that SARAF and/or Orai3 knockdown did not significantly modify the resting fura-2 fluorescence ratio (MCF7 shRNAcv: 0.33 ± 0.06 vs. siOrai3: 0.35 ± 0.06 or siOrai3+shSARAF: 0.34 ± 0.06). Interestingly, as shown in Figure 2j, the transfection of MCF7 cells with siOrai3 significantly attenuated SOCE by a similar extent than transfection of shSARAF (Figure 2d,g) or the combination of both plasmids (Figure 2j), thus suggesting that the role of SARAF in MCF7 cells is entirely mediated by Orai3 (*p* < 0.001; *n* = 40 cells/day/3–5 days). Altogether, these findings indicate that SARAF plays a negative regulatory role in SOCE in pre-neoplastic breast epithelial cells and TNBC cells. By contrast, our results indicate that SARAF plays a positive role in SOCE in ER+ breast cancer cells.

To further explore the differential role of SARAF in different breast cancer subtypes, we repeated the experiments in another ER+ and TNBC cell line, with T47D and MDA-MB-468 cells, respectively. Our results indicate that SARAF knockdown significantly enhances SOCE in T47D while suppressing SOCE in MDA-MB-468 cells, without having any effect on the resting fura-2 fluorescence ratio or Ca^2+^ release from the intracellular stores (Figure S1; *p* < 0.001; *n* = 40 cells/day/3–5 days). These findings confirm the differential role of SARAF in SOCE in ER+ and TNBC cells.

### 2.2. EFHB and SARAF Are Required for Cell Viability in Breast Cancer Cells

**Figure 3:**
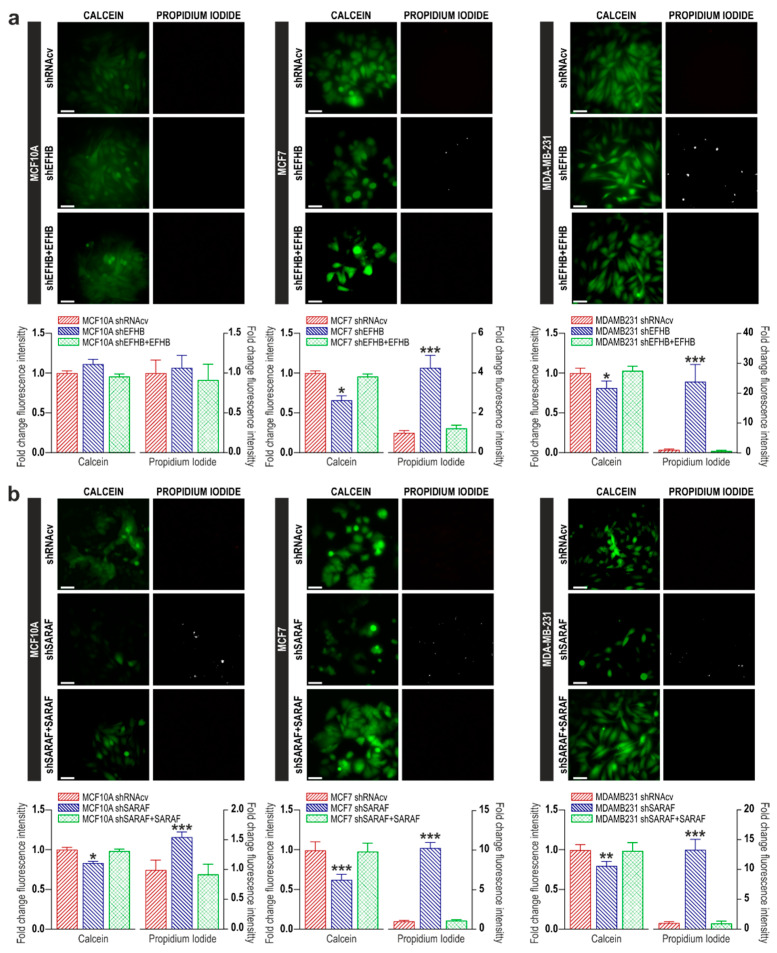

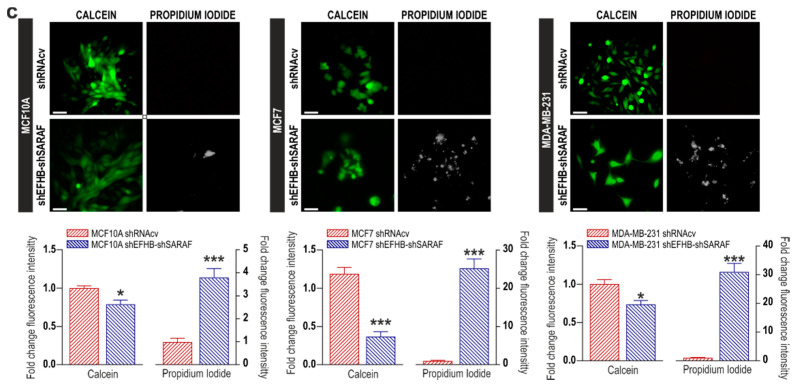
Attenuation of EFHB and SARAF expression reduces viability in breast cancer and pre-neoplastic cells. MCF10A, MCF7, and MDA-MB-231 cells were transfected with shEFHB alone or in combination of EFHB expression plasmid (**a**) shSARAF alone or in combination with SARAF expression plasmid; (**b**) shEFHB and shSARAF; (**c**) or scramble plasmids (shRNAcv), as indicated. Forty-eight hours after transfection, cells were loaded with calcein (green) and propidium iodide (white) and cell staining was visualized using an inverted microscope, as described in Material and Methods. Bar graphs represent calcein and propidium iodide staining under the different conditions expressed as fold change experimental over control (cells transfected with shRNAcv) and presented as mean ± SEM. Images shown are representative of 6–8 independent experiments. * *p* < 0.05 ** *p* < 0.01 and *** *p* < 0.001, compared to the corresponding control. Scale bar: 100 µm.

Next, we have investigated the possible role of EFHB and SARAF in cell viability in breast pre-neoplastic and neoplastic cells. To investigate this phenomenon, we have explored the effect of cell transfection with shEFHB, shSARAF, or both on cell viability by using the cell-permeant dye calcein and propidium iodide. As shown in Figure 3a, our results indicate that all MCF10A cells show that calcein fluorescence and propidium iodide staining were unaffected by EFHB knockdown (*n* = 6). However, attenuation of EFHB expression in MCF7 and MDA-MB-231 cells significantly attenuated calcein staining (*p* < 0.05; Mann–Whitney U test; *n* = 8) after 48 h. Consistently, EFHB knockdown significantly enhanced propidium iodide staining (Figure 3a; *p* < 0.05; Mann–Whitney U test; *n* = 8). These findings indicate that EFHB is specifically required for breast cancer cell viability without affecting the viability of MCF10A cells. By contrast, attenuation of SARAF expression significantly reduced the number of viable MCF10A, MCF7, and MDA-MB-231 cells (Figure 3b; *p* < 0.05; Mann–Whitney U test; *n* = 8). The combined knockdown of EFHB and SARAF did not result in an additive effect on cell viability (Figure 3c; *p* < 0.05; Mann–Whitney U test; *n* = 8), thus suggesting that EFHB and SARAF participate in the same intracellular pathway. These findings indicate that both EFHB and SARAF are essential for MCF7 and MDA-MB-231 cell viability, while only SARAF is required for MCF10A cell viability, which is consistent with the low expression of EFHB in these cells.

We have further tested whether the dependence of cell viability on SARAF and EFHB is reversible. To address this, phenomenon cells were co-transfected with shEFHB or shSARAF in combination with EFHB or SARAF expression plasmids, respectively. As shown in Figure 3a,b, overexpression of EFHB or SARAF in cells transfected with shEFHB or shSARAF reversed the effect of EFHB and SARAF knockdown in cell viability, thus indicating that the effects observed upon attenuation of EFHB and SARAF in cell viability is reversible. In addition, these findings reveal that the effects observed with the shRNA plasmids are specifically mediated by protein knockdown. 

### 2.3. EFHB and SARAF Are Required for Proliferation and Migration in Breast Cancer Cells but Not in Pre-Neoplastic Epithelial Cells

**Figure 4:**
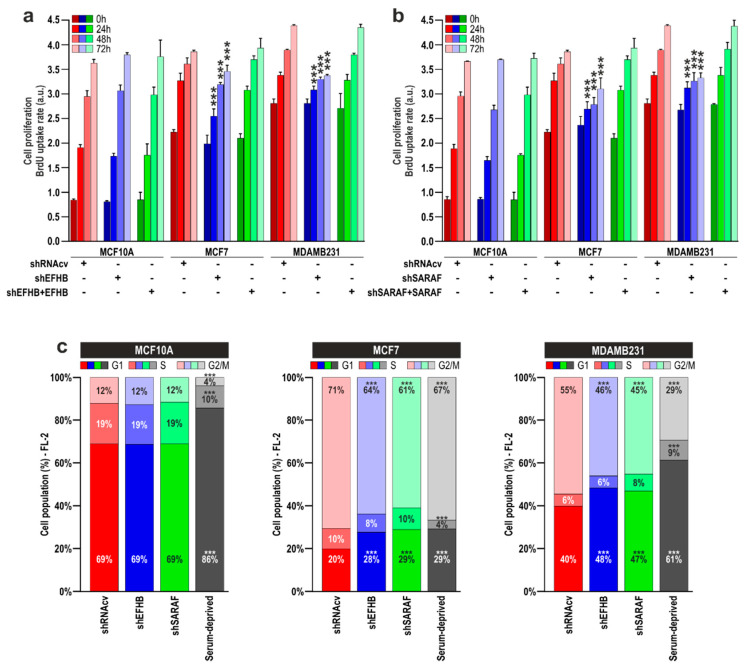
EFHB and SARAF are required for cell proliferation in breast cancer and pre-neoplastic epithelial cells. (**a**,**b**) MCF10A, MCF7, and MDA-MB-231 cells were transfected with shEFHB alone or in combination with EFHB expression plasmid (**a**) shSARAF alone or in combination with SARAF expression plasmid; (**b**) or scramble plasmids (shRNAcv as control), as indicated. Forty-eight hours after transfection, cell proliferation was assessed using the BrdU cell proliferation assay kit, as described in Material and Methods, for a further 24, 48, and 72 h. Bar graphs represent cell proliferation 0, 24, 48, and 72 h after the beginning of the experiment, presented as BrdU uptake rate. *** *p* < 0.001 compared to the corresponding control (cells transfected with shRNAcv). (**c**) MCF10A, MCF7, and MDA-MB-231 cells were transfected with shEFHB, shSARAF, or scramble plasmids (shRNAcv as control), or were serum-deprived, as indicated. Forty-eight hours later, cell cycle analysis through PI staining and following flow cytometry was performed, as described in “Materials and Methods”. Stacked bars are representative of six independent experiments.

It is well known that SOCE plays an important role in proliferation and migration in breast cancer cells [20]. Hence, we have further investigated the effect of EFHB and SARAF knockdown on the ability of MCF7 and MDA-MB-231 cells to proliferate and migrate. Cells were transfected with shEFHB, shSARAF, or scramble plasmids and, after, 48-h cell proliferation was assessed at time = 0 h, as well as 24, 48, and 72 h later. As shown in Figure 4a, EFHB knockdown was without effect on MCF10A cell proliferation, at least for the time investigated, which is compatible with the low expression of EFHB in these cells. However, attenuation of EFHB expression significantly reduced MCF7 and MDA-MB-231 cell proliferation at all the times investigated, as compared with cells transfected with scramble plasmids (*p* < 0.05 the Kruskal–Wallis test combined with Dunn’s post-hoc test; *n* = 6). These findings indicate that EFHB plays a relevant role in proliferation specifically in breast cancer cells without having a detectable role in pre-neoplastic cells. Similarly, transfection of MCF7 and MDA-MB-231 with shSARAF resulted in a significant reduction in cell proliferation compared to cells transfected with scramble plasmids (Figure 4b; *p* < 0.05 the Kruskal–Wallis test combined with Dunn’s post-hoc test; *n* = 6); however, SARAF knockdown in MCF10A did not significantly alter cell proliferation. These findings suggest that SARAF is required for breast neoplastic cell proliferation. Co-transfection of neoplastic cells with shEFHB in combination with EFHB expression plasmid completely reversed the effect of shEFHB (Figure 4a) and similar results were observed in cells transfected with shSARAF and SARAF expression plasmid (Figure 4b), which indicates that the effect of shEFHB and shSARAF transfection on cell proliferation is mediated by attenuation of the protein expression.

Thereafter, we transfected MCF10A, MCF7, and MDA-MB-231 cells with shEFHB, shSARAF, or shRNAcv, as control, and analyzed cell cycle parameters. Cells serum deprived for 24 h were used as positive control (Figure 4c). We found that MCF7 and MDA-MB-231 cells transfected with shEFHB or shSARAF displayed a G1 arrest (Figure 4c; *p* < 0.001). Similarly, serum-deprived cells also displayed a G1 arrest (Figure 4c). By contrast, EFHB or SARAF knockdown did not significantly alter MCF10A cell cycle progression (Figure 4c). These findings confirm the functional role of EFHB and SARAF in cell proliferation and cell cycle progression.

**Figure 5:**
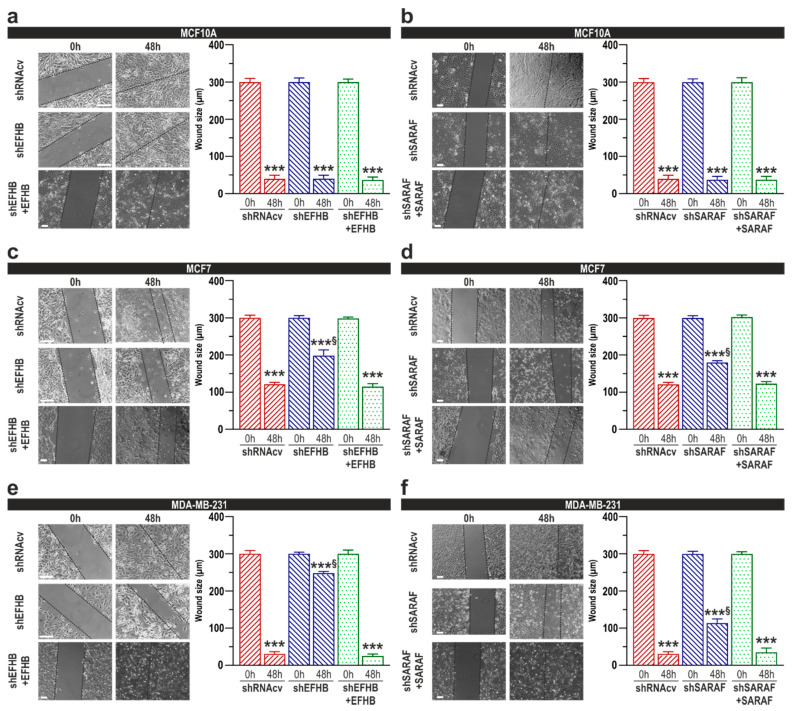
EFHB and SARAF are required for cell migration in breast cancer and pre-neoplastic epithelial cells. MCF10A (**a**,**b**), MCF7 (**c**,**d**), and MDA-MB-231 cells (**e**,**f**) were transfected with shEFHB alone or in combination with EFHB expression plasmid, shSARAF alone, or in combination with SARAF expression plasmid or scramble plasmids (shRNAcv), as indicated. Forty-eight hours after transfection cells were subjected to the wound healing assay, as described in Methods. Images were acquired at 0 and 48 h from the beginning of the assay. The dotted lines define the areas lacking cells. The bar graphs represent the wound size, in micrometers, at the different conditions, expressed as the mean ± SEM of six independent experiments. *** *p* < 0.001 compared to the time = 0 h. ^§^ *p* < 0.001 compared to the corresponding time in shRNAcv-transfected cells. Scale bar: 50 µm.

We have also assessed the functional role of EFHB and SARAF on MCF10A, MCF7, and MDA-MB-231 cells migration using the well-established wound healing assay [21]. Cells transfected with scramble plasmids significantly reduced the wound size during the first 48 h (Figure 4c; *p* < 0.05 Mann–Whitney U test; *n* = 6). Transfection of MCF10A cells with shEFHB did not significantly impair cell migration at least for 48 h (Figure 5a; *n* = 6), which is attributed to the low EFHB expression in these cells. By contrast, EFHB knockdown significantly attenuated cell migration in MCF7 and MDA-MB-231 cells (Figure 5c,e; *p* < 0.001; *n* = 6), thus suggesting that EFHB plays a relevant role in MCF7 and MDA-MB-231 cells migration without affecting the ability of MCF10A cells to migrate. Similarly, a reduction in SARAF expression had no impact in MCF10A cells migration (Figure 5b; *n* = 6), whereas SARAF knockdown significantly impaired cell migration in MCF7 and MDA-MB-231 cells (Figure 5d,f; *p* < 0.001 Mann–Whitney U test; *n* = 6). All together, our data suggest that SARAF function is important for MCF7 and MDA-MB-231 cells migration. As mentioned above, co-transfection with EFHB or SARAF expression plamids revesed the inhibitory effect of EFHB and SARAF knockdown in cell migration (Figure 5c–f).

## 3. Discussion

**Figure 6:**
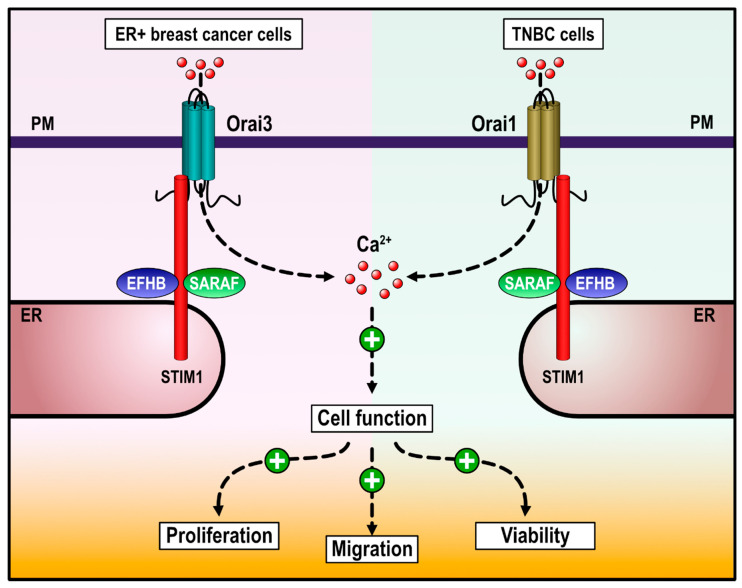
Cartoon summarizing the functional role of SARAF and EFHB in ER+ and triple negative breast cancer (TNBC) cells. In ER+ breast cancer cells and TNBC cells, SOCE is predominantly mediated by Orai3 and Orai1 channels, respectively. SARAF and EFHB modulate STIM1 activation and function and fine-tune SOCE, resulting in adequate Ca^2+^ signals for cell proliferation, migration, and viability.

SOCE has been reported to play an essential role in the development of a variety of breast cancer hallmarks, including cell viability, migration, proliferation, and apoptosis resistance [22,23,24,25]. STIM1 and Orai1 are the key elements in SOCE but several proteins, such as SARAF and EFHB, play an important role fine-tuning the amount of Ca^2+^ entering the cell through the store-operated channels [13,14,16,17]. Here, we report that EFHB is overexpressed in ER+ and TNBC cells compared to pre-neoplastic cells while neoplastic and pre-neoplastic cells exhibit a similar expression of SARAF. In addition, both proteins play an important role in the activation of SOCE in breast cancer cells. Our results further indicate that EFHB and SARAF are required for breast cancer MCF7 and MDA-MB-231 cells viability, proliferation, and migration (Figure 6), although the signaling pathway might differ between both cell types. In MCF7 cells, SARAF and EFHB play a positive effect on SOCE, while in MDA-MB-231 cells, the SARAF and EFHB signalplex plays a regulatory role in SOCE, thus preventing Ca^2+^ overload, as previously described in other cellular models [13,14,26,27].

Our findings reveal that SARAF knockdown significantly reduces SOCE in the ER+ breast cancer MCF7 cell line. To our knowledge, this is the first description of a predominant positive role of SARAF in SOCE in a given cell type, but in a recent study we have reported that SARAF and Orai1 are required for Ca^2+^ influx induced by VEGF in vascular endothelial cells [18]. SOCE in MCF7, as well as in other ER+ breast cancer cell lines, exhibits significant phenotypic and functional differences compared to other cell types. For instance, while the expression of STIM1 in these cells is normal or low, STIM2 expression is slightly higher, and both Orai1 and Orai3 are overexpressed, especially Orai3 [9,10,28]. From the functional point of view, SOCE in MCF7 is mediated by STIM1, STIM2, and Orai3, in contrast to other breast cancer subtypes, such as TNBC cells, where SOCE is entirely dependent on STIM1 and Orai1 [9,10]. In addition to its role in SOCE, STIM2 also plays a relevant role in the maintenance of resting cytosolic and ER Ca^2+^ concentration in MCF7 cells [28]. SARAF has been described as a negative modulator of STIM1 and STIM2 but its possible role on Orai3 function has not been elucidated. SARAF interacts with STIM1 at rest, to prevent STIM1 spontaneous activation [13], and mediates STIM1 deoligomerization upon Ca^2+^ store refilling, finally leading to SCDI [13,14]. Concerning STIM2, in HEK-293T cells, SARAF modulates resting cytosolic and ER Ca^2+^ concentrations by inhibition of STIM2-dependent basal SOCE [13]. Here, we show that knockdown of Orai3, SARAF, or both in MCF7 cells results in a similar degree of inhibition of SOCE, which might indicate that SARAF is necessary for the activation of Orai3 and thus SOCE in MCF7 cells, and suggests that SARAF plays a positive role on Orai3 channel function. 

Our results indicate that EFHB and SARAF are expressed in neoplastic and pre-neoplastic cells. However, while the expression of SARAF is similar in breast cancer and pre-neoplastic cells, EFHB expression is significantly greater in cancer cells. In a model where SARAF plays an inhibitory role in STIM1 function [13,14,16,29] and EFHB transiently releases STIM1 from SARAF, thus facilitating SOCE [19], one would expect that, if SARAF is similarly expressed in cells of a given lineage, the expression of EFHB could make a difference between the extent of SOCE in those cell types. This might be the case for MCF10A, which exhibits the lowest EFHB expression and the smaller extent of SOCE compared to MDA-MB-231 cells. Although we cannot rule out other functional differences between these cell types, the relative different expression of EFHB might be, at least partially, involved in the different extent of SOCE observed among the cells investigated.

## 4. Materials and Methods 

### 4.1. Materials and Reagents 

Fura-2 acetoxymethyl ester (fura-2/AM) was purchased from Molecular Probes (Leiden, The Netherlands). Thapsigargin (TG), a rabbit polyclonal anti-β-actin antibody (catalog number A2066, epitope: amino acids 365–375 of human β-actin), bovine serum albumin (BSA), insulin (catalog number: I9278), epidermal growth factor (catalog number: E9644), esiOrai3 (a pool of siRNAs that target the Orai3 mRNA), and cholera toxin (catalog number: C8052) were purchased from Sigma-Aldrich (Madrid, Spain). A rabbit polyclonal anti-TMEM66 (SARAF) antibody (catalog number PA5-24237, epitope amino acids 33–62 of the N-terminal region of human TMEM66), and a Live/Dead^®^ viability/cytotoxicity kit were purchased from Thermo Fisher (Madrid, Spain). DharmaFECT was purchased from Dharmacon (Lafayette, CO, USA). A rabbit polyclonal anti-EFHB antibody (catalog number AP10904a, epitope: amino acids 1–30 of human EFHB (N-terminal)) was purchased from Abgent (San Diego, CA, USA). A horseradish peroxidase-conjugated anti-rabbit IgG antibody was purchased from Jackson (Cambridge, UK). Enhanced chemiluminescence detection reagents were from Pierce (Cheshire, UK). A bromodeoxyuridine (BrdU) cell proliferation assay kit was purchased from BioVision (Milpitas, CA, USA). All other reagents were of analytical grade. 

### 4.2. Cell Culture and Transfections

The human breast epithelial MCF10A cell line was provided by Dr. Potier-Cartereau (Université François Rabelais Tours, France). The breast cancer MCF7 and MDA-MB-231 cell lines were obtained from ATCC (Manassas, VA, USA), and cultured at 37 °C with a 5% CO_2_ in DMEM-F12 (MCF10A) or DMEM (MCF7 and MDA-MB-231), supplemented with 10% (*v*/*v*) horse or fetal bovine serum, respectively, and 100 U/mL of penicillin and streptomycin.

For the knockdown expression of SARAF, a pLKO.1-puro plasmid-based shRNA targeting the sequence CGGACTTAGATATTGCATACA (clone ID: TRCN0000146643; Sigma-Aldrich) was used [16]. For the knockdown expression of EFHB, a pLKO.1-puro plasmid-based shRNA targeting the sequence GCCAGAAGATATTGTCTTAAA (clone ID: TRCN0000055578; Sigma-Aldrich) was used [19]. Cells were transfected with the shEFHB, shSARAF, esiOrai3, or scramble plasmids, as described previously [16,19] using DharmaFECT transfection reagent, and were used 48 h after transfection. Plasmids were used for silencing experiments at 1 µg/mL.

### 4.3. Determination of Cytosolic Free-Ca^2+^ Concentration

Cells were loaded with fura-2 by incubation with 5 μM of fura 2/AM (MCF10A) or 2 µM of fura 2/AM (MCF7 and MDA-MB-231) for 30 min at 37 °C. Coverslips with attached cultured cells were mounted on a perfusion chamber and, subsequently, placed on the stage of an epifluorescence inverted microscope (Nikon Eclipse Ti2, Amsterdam, The Netherlands) with image acquisition and analysis software (NIS-Elements Imaging Software v.5.02.00, Nikon). Cells were superfused with HEPES-buffered saline (HBS) containing (in mM) 125 NaCl, 5 KCl, 1 MgCl_2_, 5% glucose, 25 HEPES, and pH 7.4, supplemented with 0.1% (*w*/*v*) BSA. Samples were alternatively excited with light from a xenon lamp passed through a high-speed monochromator (Optoscan ELE 450, Cairn Research, Faversham, UK) at 340/380 nm. Fluorescence emission at 505 nm was detected using a sCMOS camera (Zyla 4.2, Andor, Belfast, UK) and recorded using NIS-Elements AR software (Nikon, Amsterdam, The Netherlands). A fluorescence ratio (F340/F380) was calculated pixel by pixel [21] and was normalized to the ratio in resting cells (F/F_0_). TG-evoked Ca^2+^ release and SOCE were measured as the integral of the rise in the fura-2 fluorescence ratio for 5 and 2½ min, respectively, after the addition of TG or Ca^2+^, respectively.

### 4.4. Western Blotting

Western blotting was performed, as described previously. Briefly, cell lysates were resolved by 10% SDS-PAGE and separated proteins were electrophoretically transferred onto nitrocellulose membranes for subsequent probing. Blots were incubated overnight with 10% (*w*/*v*) BSA in Tris-buffered saline with 0.1% Tween 20 (TBST) to block residual protein binding sites. Immunodetection of EFHB, SARAF, and β-actin was achieved by incubation overnight with the anti-SARAF or anti-EFHB antibodies diluted 1:1000 in TBST, or by incubation for 1 h with an anti-β-actin antibody diluted 1:2000 in TBST. The primary antibody was removed, and blots were washed six times for 5 min each with TBST. To detect the primary antibody, blots were incubated for 1 h with horseradish peroxidase-conjugated goat anti-rabbit IgG antibody diluted 1:10,000 in TBST and then exposed to enhanced chemiluminescence reagents for 5 min. The density of bands was measured using C-DiGit Chemiluminescent Western Blot Scanner and Fiji-ImageJ software (NIH, Bethesda, MD, USA).

### 4.5. Cell Proliferation

Cells were seeded at a concentration of 5 × 10^3^/well (MCF7 and MDA-MB-231) or 1 × 10^3^/well (MCF10A) into 96-well plates and, after 0, 24, 48, and 72 h, cell proliferation was assessed using a specific cell proliferation assay kit based on the measurement of BrdU incorporation during DNA synthesis according to the manufacturer’s instructions (BioVision). Absorbance in samples was measured using a plate reader (Epoch, Biotek, Swindon, UK) at 450 nm and presented as arbitrary units.

### 4.6. Cell Viability Assay

Cell viability was assessed using a Live/Dead^®^ viability/cytotoxicity kit as described previously [30]. Briefly, cells were incubated for 45 min with 2 μM of calcein-AM and 4 μM of propidium iodide, as per the manufacturer’s instructions. Cells were washed and resuspended in fresh HBS. Coverslips with attached cultured cells were mounted on a perfusion chamber and placed on the stage of an epifluorescence inverted microscope (Nikon Eclipse Ti-2) with an image acquisition and analysis system (Nikon NIS-Elements AR v.5.02.00). Samples were excited at 430 nm and 555 nm for calcein and propidium iodide, respectively, and the resulting fluorescence was recorded at 542 nm (for viable cells) and 624 nm (for dead cells).

### 4.7. Wound-Healing Assay

For wound healing assay, MCF10A, MCF7, and MDA-MB-231 cells were seeded in a 35-mm 6-well multidish to obtain confluence after 24 h. Next, cells were cultured in medium supplemented with 1% serum, and a wound was created using a sterile 200-µL plastic pipette tip. Photographs were taken immediately or at the times indicated using an EVOS FL auto 2 cell imaging system (ThermoFisher Scientific, Madrid, Spain). Migration of cells was quantitated using Fiji ImageJ (NIH, Bethesda, MD, USA).

### 4.8. Determination of Cell Cycle by Flow Cytometry

Cell cycle was evaluated using propidium iodide (PI). Then, 48 h after transfection or 24 h after serum deprivation, 2 × 10^6^ cells were fixed with EtOH 70% at −20 °C for 12–24 h. Next, EtOH was removed and washed twice with fresh ice-cold PBS and the cells were permeabilized with PBS containing 0.1% (*v*/*v*) Triton X-100 and 100 µg/mL RNAasa at 37 °C for 15–30 min. The percentages of cell cycle stages were defined by incubating propidium iodide (2 µM) at 37 °C for 30 min in darkness, as per the manufacturer’s instructions. To analyze cell cycle status, cellular DNA content was estimated using a FACS-SCAN flow cytometer (Becton Dickinson, Madrid, Spain). Forward and side scattering were considered to select appropriated cells. The fluorescence emitted from cells was acquired at a wavelength of 555/624 nm (Ex/Em) for PI.4.9. 

Analysis of statistical significance was performed using the Kruskal–Wallis test combined with Dunn’s post-hoc test (GraphPad Prism Windows 8, San Diego, CA, USA). For comparison between two groups, the Mann–Whitney U test (or Student’s *t*-test for the analysis of Ca^2+^ determinations) was used. A *p*-value < 0.05 was considered to be statistically significant.

## 5. Conclusions

Our results indicate that SARAF and EFHB are expressed in breast cancer and pre-neoplastic breast epithelial cells and play a relevant role in SOCE. While SARAF expression is similar in neoplastic and pre-neoplastic cells, EFHB is overexpressed in breast cancer cells, which might partially explain the enhanced SOCE observed in cancer cells. Cell viability, proliferation, and migration is strongly dependent on SARAF and EFHB, or exclusively SARAF, in breast cancer and pre-neoplastic cells, respectively, thus suggesting that these proteins play an important role in breast cancer development and progression.

## Data Availability

The data presented in this study are available on request from the corresponding author.

## Supplementary Materials

The following are available online at https://www.mdpi.com/article/10.3390/cancers13164160/s1, Figure S1: Functional role of SARAF in Ca^2+^ release and SOCE in the ER+ T47D and TNBC MDA-MB-468 cell lines, Figure S2: Uncropped western blot figures of Figure 1a,b and Figure 2a,b.

## References

[1] Kappel S., Borgstrom A., Stoklosa P., Dorr K., Peinelt C. (2019). Store-operated calcium entry in disease: Beyond STIM/Orai expression levels. Semin. Cell. Dev. Biol..

[2] Smani T., Shapovalov G., Skryma R., Prevarskaya N., Rosado J.A. (2015). Functional and physiopathological implications of TRP channels. Biochim. Biophys. Acta.

[3] Hou X., Pedi L., Diver M.M., Long S.B. (2012). Crystal structure of the calcium release-activated calcium channel Orai. Science.

[4] Yoast R.E., Emrich S.M., Zhang X., Xin P., Johnson M.T., Fike A.J., Walter V., Hempel N., Yule D.I., Sneyd J. (2020). The native ORAI channel trio underlies the diversity of Ca^2+^ signaling events. Nat. Commun..

[5] Roos J., DiGregorio P.J., Yeromin A.V., Ohlsen K., Lioudyno M., Zhang S., Safrina O., Kozak J.A., Wagner S.L., Cahalan M.D. (2005). STIM1, an essential and conserved component of store-operated Ca^2+^ channel function. J. Cell. Biol..

[6] Zhang S.L., Yu Y., Roos J., Kozak J.A., Deerinck T.J., Ellisman M.H., Stauderman K.A., Cahalan M.D. (2005). STIM1 is a Ca^2+^ sensor that activates CRAC channels and migrates from the Ca^2+^ store to the plasma membrane. Nature.

[7] Berna-Erro A., Braun A., Kraft R., Kleinschnitz C., Schuhmann M.K., Stegner D., Wultsch T., Eilers J., Meuth S.G., Stoll G. (2009). STIM2 regulates capacitive Ca^2+^ entry in neurons and plays a key role in hypoxic neuronal cell death. Sci. Signal..

[8] Berna-Erro A., Jardin I., Salido G.M., Rosado J.A. (2017). Role of STIM2 in cell function and physiopathology. J. Physiol..

[9] Motiani R.K., Abdullaev I.F., Trebak M. (2010). A novel native store-operated calcium channel encoded by Orai3: Selective requirement of Orai3 versus Orai1 in estrogen receptor-positive versus estrogen receptor-negative breast cancer cells. J. Biol. Chem..

[10] Jardin I., Diez-Bello R., Lopez J.J., Redondo P.C., Salido G.M., Smani T., Rosado J.A. (2018). TRPC6 Channels Are Required for Proliferation, Migration and Invasion of Breast Cancer Cell Lines by Modulation of Orai1 and Orai3 Surface Exposure. Cancers.

[11] Yang S., Zhang J.J., Huang X.Y. (2009). Orai1 and STIM1 are critical for breast tumor cell migration and metastasis. Cancer Cell..

[12] McAndrew D., Grice D.M., Peters A.A., Davis F.M., Stewart T., Rice M., Smart C.E., Brown M.A., Kenny P.A., Roberts-Thomson S.J. (2011). ORAI1-mediated calcium influx in lactation and in breast cancer. Mol. Cancer Ther..

[13] Palty R., Raveh A., Kaminsky I., Meller R., Reuveny E. (2012). SARAF inactivates the store operated calcium entry machinery to prevent excess calcium refilling. Cell.

[14] Jha A., Ahuja M., Maleth J., Moreno C.M., Yuan J.P., Kim M.S., Muallem S. (2013). The STIM1 CTID domain determines access of SARAF to SOAR to regulate Orai1 channel function. J. Cell. Biol..

[15] Albarran L., Lopez J.J., Woodard G.E., Salido G.M., Rosado J.A. (2016). Store-operated Ca^2+^ entry-associated regulatory factor (SARAF) plays an important role in the regulation of arachidonate-regulated Ca^2+^ (ARC) channels. J. Biol. Chem..

[16] Albarran L., Lopez J.J., Ben Amor N., Martín-Cano F.E., Berna-Erro A., Smani T., Salido G.M., Rosado J.A. (2016). Dynamic interaction of SARAF with STIM1 and Orai1 to modulate store-operated calcium entry. Sci. Rep..

[17] Albarran L., Lopez J.J., Gomez L.J., Salido G.M., Rosado J.A. (2016). SARAF modulates TRPC1, but not TRPC6, channel function in a STIM1-independent manner. Biochem. J..

[18] Galeano-Otero I., Del Toro R., Khatib A.M., Rosado J.A., Ordonez-Fernandez A., Smani T. (2021). SARAF and Orai1 Contribute to Endothelial Cell Activation and Angiogenesis. Front. Cell. Dev. Biol..

[19] Albarran L., Lopez J.J., Jardin I., Sanchez-Collado J., Berna-Erro A., Smani T., Camello P.J., Salido G.M., Rosado J.A. (2018). EFHB is a Novel Cytosolic Ca^2+^ Sensor That Modulates STIM1-SARAF Interaction. Cell. Physiol. Biochem..

[20] Azimi I., Bong A.H., Poo G.X.H., Armitage K., Lok D., Roberts-Thomson S.J., Monteith G.R. (2018). Pharmacological inhibition of store-operated calcium entry in MDA-MB-468 basal A breast cancer cells: Consequences on calcium signalling, cell migration and proliferation. Cell. Mol. Life Sci..

[21] Jardin I., Diez-Bello R., Falcon D., Alvarado S., Regodon S., Salido G.M., Smani T., Rosado J.A. (2020). Melatonin downregulates TRPC6, impairing store-operated calcium entry in triple negative breast cancer cells. J. Biol. Chem..

[22] Gueder N., Allan G., Telliez M.S., Hague F., Fernandez J.M., Sanchez-Fernandez E.M., Ortiz-Mellet C., Ahidouch A., Ouadid-Ahidouch H. (2017). sp(2) -Iminosugar alpha-glucosidase inhibitor 1-C-octyl-2-oxa-3-oxocastanospermine specifically affected breast cancer cell migration through Stim1, beta1-integrin, and FAK signaling pathways. J. Cell. Physiol..

[23] Sanchez-Collado J., Lopez J.J., Jardin I., Camello P.J., Falcon D., Regodon S., Salido G.M., Smani T., Rosado J.A. (2019). Adenylyl Cyclase Type 8 Overexpression Impairs Phosphorylation-Dependent Orai1 Inactivation and Promotes Migration in MDA-MB-231 Breast Cancer Cells. Cancers.

[24] Lopez J.J., Siegfried G., Cantonero C., Soulet F., Descarpentrie J., Smani T., Badiola I., Pernot S., Evrard S., Rosado J.A. (2021). Furin Prodomain ppFurin Enhances Ca^2+^ Entry Through Orai and TRPC6 Channels’ Activation in Breast Cancer Cells. Cancers.

[25] Kulkarni R.P., Elmi A., Alcantara-Adap E., Hubrack S., Nader N., Yu F., Dib M., Ramachandran V., Najafi Shoushtari H., Machaca K. (2019). miRNA-dependent regulation of STIM1 expression in breast cancer. Sci. Rep..

[26] Jardin I., Albarran L., Salido G.M., Lopez J.J., Sage S.O., Rosado J.A. (2018). Fine-tuning of store-operated calcium entry by fast and slow Ca^2+^-dependent inactivation: Involvement of SARAF. Biochim. Biophys. Acta. Mol. Cell. Res..

[27] Lopez E., Frischauf I., Jardin I., Derler I., Muik M., Cantonero C., Salido G.M., Smani T., Rosado J.A., Redondo P.C. (2019). STIM1 phosphorylation at Y(316) modulates its interaction with SARAF and the activation of SOCE and I CRAC. J. Cell. Sci..

[28] Sanchez-Collado J., Lopez J.J., Gonzalez-Gutierrez L., Cantonero C., Jardin I., Salido G.M., Rosado J.A. (2020). Functional role of TRPC6 and STIM2 in cytosolic and endoplasmic reticulum Ca^2+^ content in resting estrogen receptor-positive breast cancer cells. Biochem. J..

[29] Albarran L., Regodon S., Salido G.M., Lopez J.J., Rosado J.A. (2016). Role of STIM1 in the surface expression of SARAF. Channels.

[30] Diez-Bello R., Jardin I., Lopez J.J., El Haouari M., Ortega-Vidal J., Altarejos J., Salido G.M., Salido S., Rosado J.A. (2019). (–)-Oleocanthal inhibits proliferation and migration by modulating Ca^2+^ entry through TRPC6 in breast cancer cells. Biochim. Biophys. Acta Mol. Cell. Res..

