# Supplementary material for: SARAF and EFHB Modulate Store-Operated Ca2+ Entry and Are Required for Cell Proliferation, Migration and Viability in Breast Cancer Cells
Source: Cancers (Basel). 2021 Aug 19;13(16):4160. doi: 10.3390/cancers13164160 (PMC8393677; doi:10.3390/cancers13164160)
Supplement: Supplementary file 1 [file cancers-13-04160-s001.zip › cancers-1285363-supplementary.pdf]

Supplementary Materials: SARAF and EFHB Modulate Store-Operated Ca²⁺ Entry and are Required for Cell Proliferation, Migration and, Viability in Breast Cancer Cells

Isaac Jardin Joel Nieto, Sandra Alvarado, Raquel Diez-Bello, Jose J. Lopez, Ginés M. Salido, Tarik Smani and Juan A. Rosado

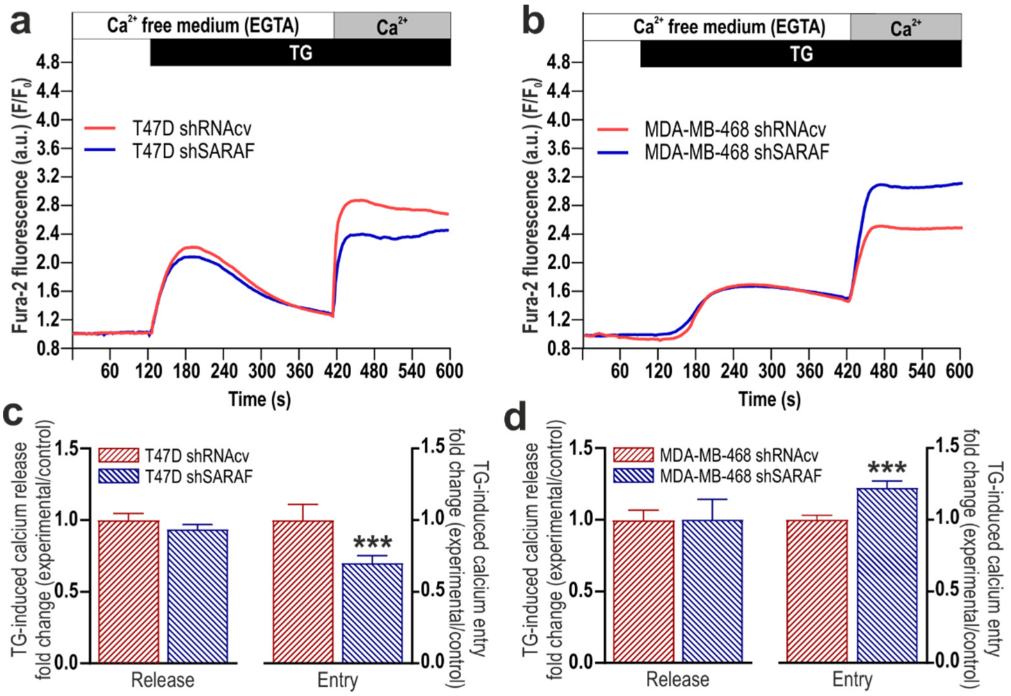

Figure S1. Functional role of SARAF in Ca^{2+} release and SOCE in the ER+ T47D and TNBC MDA-MB-468 cell lines. T47D (**a**) and MDA-MB-468 cells (**b**) were transfected with shSARAF or scramble plasmids (shRNAcv), as indicated. Forty-eight hours after transfection, cells were loaded with fura-2 and perfused with a Ca^{2+}-free medium (100 µM EGTA added). Cells were then stimulated with TG (2 µM) followed by reintroduction of external Ca^{2+} (final concentration 1 mM) to initiate Ca^{2+} entry. Bar graphs represent TG-induced Ca^{2+} release and entry in T47D (**c**) and MDA-MB-468 (**d**), expressed as fold change over control (shRNAcv-treated cells). Data are mean ± SEM of 40 cells/day/3–5 days. *** $p < 0.001$ compared to Ca^{2+} entry in control cells.

figure 1a

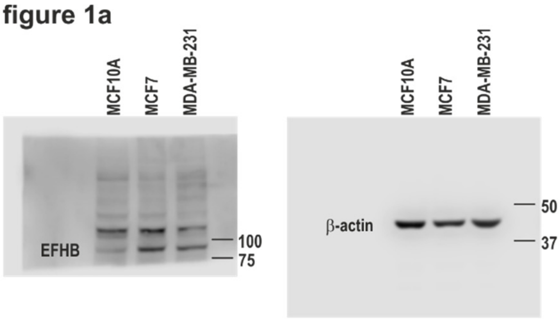

	EFHB	ACTIN	RATIO
MCF10A	7938.569	15691.033	0.5059303
MCF7	13776.447	11719.033	1.17556175
MDAMB231	10207.548	12345.326	0.82683503

figure 1b

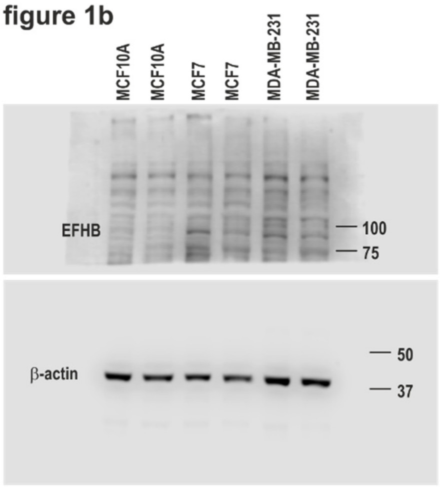

	EFHB	ACTIN	RATIO
MCF10A	7704.033	12224.962	0.63018871
MCF10A shEFHB	7841.761	12275.79	0.63879889
MCF7	14288.225	10841.497	1.31791993
MCF7 shEFHB	10326.296	11100.64	0.9302433
MDAMB231	15340.56	17215.154	0.89110792
MDAMB231 shEFHB	10173.296	16866.054	0.60318175

figure 2a

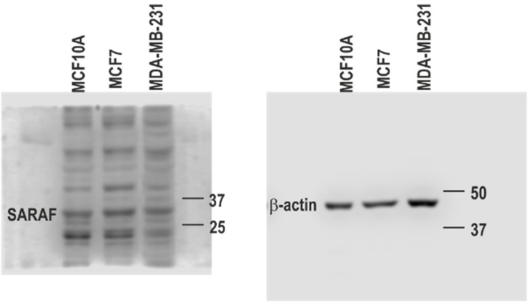

	SARAF	ACTIN	RATIO
MCF10A	25871.38	24913.267	1.0384579429105
MCF7	31850.723	28762.53	1.10736861465247
MDAMB231	27369.874	32836.238	0.833526483758584

figure 2b

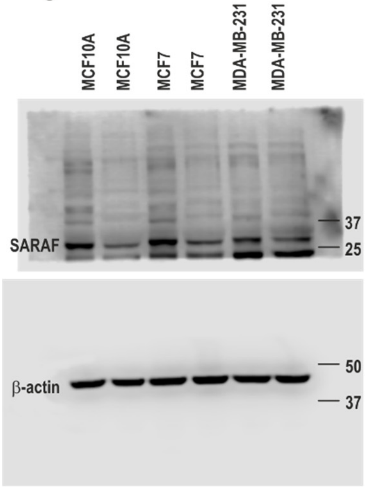

	SARAF	ACTIN	RATIO
MCF10A	19890.69	20904.79	0.95149
MCF10A shEFHB	13112.983	20683.376	0.63399
MCF7	23992.104	22339.326	1.07399
MCF7 shEFHB	15994.347	22843.861	0.70016
MDAMB231	18200.418	20294.255	0.89683
MDAMB231 shEFHB	11270.062	20022.569	0.56287

Figure S2. Uncropped western blot figures of Figure 1a, 1b, 2a and 2b.
